# Association between sleep state misperception and bedtime behavior in patients with chronic insomnia

**DOI:** 10.1038/s41598-024-64355-3

**Published:** 2024-06-18

**Authors:** Mizuki Kudo, Naoko Ayabe, Masahiro Takeshima, Masaya Ogasawara, Yu Itoh, Kazuhisa Yoshizawa, Shingo Kitamura, Kazuo Mishima

**Affiliations:** 1 Department of Neuropsychiatry, Akita University Graduate School of Medicine, 1-1-1 Hondo, Akita City, 010-8543 Japan; 2 Department of Regional Studies and Humanities, Faculty of Education and Human Studies, Akita University, 1-1 Tegata-Gakuenmachi, Akita, 010-8502 Japan; 3 Department of Sleep-Wake Disorders, National Center of Neurology and Psychiatry, National Institute of Mental Health, Tokyo, Japan

**Keywords:** Circadian rhythms and sleep, Wakefulness

## Abstract

Previous studies on sleep state misperception have objectively evaluated sleep status in special environments using polysomnography. There is a paucity of data from studies that evaluated habitual sleep status in home environments. The present study aimed to investigate sleep state misperception in the home environment of patients with chronic insomnia using a lumbar-worn actigraphy to identify sleep habits associated with sleep state misperception severity. Thirty-one patients and 42 healthy volunteers were included in the insomnia and non-insomnia group, respectively. Participants recorded subjective assessments in sleep diaries, objective assessments with an actigraphy worn for 14 days, and self-assessments using questionnaires. Both groups had similar objective sleep ratings; however, insomnia group had significantly worse subjective ratings (total sleep time, wake after sleep onset, and sleep onset latency). A significant correlation was found between subjective and objective total sleep time scores in non-insomnia group but not in insomnia group. Insomnia group had earlier bedtimes, significantly longer bedtimes, and impaired daytime functioning (Sheehan Disability Scale score); additionally, they underestimated their total sleep time, particularly with earlier bedtimes and longer laying durations. Monitoring the sleep status and habits of individuals in home environments could be instrumental in identifying key points for targeted interventions on sleep hygiene and cognitive behavioral therapy for insomnia.

## Introduction

In the general population, 6–10% of adults meet the diagnostic criteria for insomnia, making it one of the most common sleep–wake disorders^1,2^. The International Classification of Sleep Disorders (ICSD-3)^3^ and the Diagnostic & Statistical Manual of Mental Disorders (DSM-5)^4^, which are representative international diagnostic criteria for sleep–wake disorders, define insomnia as "dissatisfaction with sleep quality and quantity despite adequate opportunity to sleep" without requiring objective sleep indicators for diagnosis. However, there is often a discrepancy between subjective and objective sleep assessments in patients, with a widely recognized tendency to underestimate total sleep time (TST), overestimate sleep onset latency (SOL), and overestimate wake after sleep onset (WASO) compared to healthy individuals^5–7^. The ICSD introduced the concept of sleep state misperception (SSM) for this condition, and its notable manifestation is paradoxical insomnia^8^. Recent studies suggest that SSM occurs in primary insomnia as well as a wide range of other sleep disorders. SSM can be clinically challenging, leading to increased medication use and increased risk of falls, fractures, and cognitive impairment^9,10^.

Previous SSM studies have often measured objective sleep states using polysomnography (PSG). Although PSG is the standard method for objective sleep assessment, it tends to measure sleep states under unnatural conditions because of the need to wear different sensors and sleep in a specialized sleep environment. Moreover, it is limited by short-term measurements over a few days. Consequently, this may introduce measurement biases such as the first-night effect^13–15^. Therefore, to more accurately investigate the reality of SSM, it is preferable to assess natural sleep in home settings.

Actigraphy enables the evaluation of sleep/wake states in home settings. This device has various types, including wrist-worn and lumbar-worn models. Each model is fitted with an algorithm for determining sleep and wakefulness based on body movement data measured over time, mainly using accelerometers. Although its accuracy varies from instrument to instrument and is not in perfect agreement with PSG, it is simpler and less burdensome for the individual, making continuous measurements easier^16^. There have been several previous studies on SSM using PSG; however, to our knowledge, few have examined the real state of SSM in a home setting^17^. Furthermore, sleep SSM may be influenced by bed habits^18,19^, which are often observed in insomnia, and home-based investigations may be advantageous in this regard. Therefore, this study aims to examine the actual state of SSM among patients with chronic insomnia in the home environment and to clarify the characteristics of sleep habits that are associated with the severity of SSM.

## Methods

### Ethical considerations

This study was conducted according to the principles of the Declaration of Helsinki. This study was approved by the Ethics Committees of the National Center of Neurology and Psychiatry and Akita University School of Medicine and informed consent was received from each participant.

### Participants

The insomnia group included patients who were diagnosed with primary insomnia according to the Diagnostic & Statistical Manual of Mental Disorders, fifth edition (DSM-5) and attended an outpatient clinic for sleep disorders where the co-researcher practices. All patients were also receiving pharmacological treatment. The non-insomnia group comprised community-living adults who did not have insomnia symptoms and were recruited through flyers, posters, and the internet. A psychiatrist verified through an interview that the participants had not taken any sleep medications, including over-the-counter drugs, and had not been diagnosed with any psychiatric disorder, including primary insomnia. They were fully informed of the aims and objectives of the research. Subsequently, written informed consent was provided by all participants.

### Procedure

Participants tracked their sleep for two weeks using a self-report sleep diary and an actigraphy. They wore an actigraphy on their waist for 24 h, except when bathing and during strenuous physical activity. Additionally, they were instructed to maintain a daily sleep diary. Furthermore, a self-administered questionnaire was used to collect demographic data (age, gender), medical history, insomnia status (Athens Insomnia Scale [AIS])^20,21^, daytime functional impairment (Sheehan Disability Scale [SDISS])^22,23^, and sleep status (Pittsburgh Sleep Quality Index [PSQI])^24,25^.

### Sleep diary

Participants kept a sleep diary each morning, recording the time they turned off the lights, fell asleep, woke up during the night, last woke, and got up in the morning. Data collected from the diary were used to calculate the total time in bed (TIB), subjective TST (sj-TST), subjective SOL (sj-SOL), and subjective WASO (sj-WASO).

### Actigraphy

Participants wore an actigraphy, either MTN240 (ACOS CO., LTD, Iida city, Japan) or FS-760 (ACOS CO., LTD. Iida city, Japan), on their waist for 24 h (except when bathing and during strenuous exercise) to record the objective sleep conditions. The MTN240 is a small, lightweight, button-type device with a diameter of 27 mm that uses a built-in 3-axis acceleration sensor (a capacitance sensor) to record the amount of activity. It detects acceleration above a certain level and records the times it exceeds a threshold value during a 2-min period. The FS-760 is a rectangular, lumbar-worn device that has similar features and performance to the MTN240, although it is different in shape and size. The algorithm was validated in a previous study by comparing it with PSG^26^. The activity data obtained were analyzed with the analysis software Sleep-Sign-Act (Kissei Comtec, Matsumoto, Japan).

In this study, the objective TST (oj-TST), SOL (oj-SOL), and WASO (oj-WASO) were calculated from the recorded activity using the inherent analysis algorithm employed in a previous study^26^.

### Questionnaires

#### Athens insomnia scale

The AIS is an 8-question survey^20,21^ that includes questions regarding the difficulty in falling asleep, waking mid-way, waking early in the morning, total sleep time, sleep quality, and the impact of these attributes during daytime. Each question is rated on a scale of 0–3, with a total score calculated. The total score ranged from 0 to 24. A person is considered symptomatic if he or she has experienced sleep disturbance at least three times a week in the past month. A score of ≥ 4 is classified as "suspected insomnia," and a score of ≥ 6 as "probable insomnia.” The Japanese version of the questionnaire was used in this study. Its validity and reliability has been demonstrated in a previous study^20^.

#### Sheehan disability scale

The SDISS is a self-report questionnaire that assesses the degree of functional impairment in three main areas of daily living: work and school, home life and household chores, and social and leisure activities that subjects have experienced in the past seven days^22,23^. Each segment is rated on a scale of 0 (not at all) to 10 (very severe). The individual scores are summed to compute an overall score ranging from 0 to 30. The cutoff value is a score of ≥ 5 for any individual scores, and the overall score is used as the outcome. The Japanese version of SDISS was used in this study. Its validity and reliability have been demonstrated in a previous study^23^.

#### Pittsburgh sleep quality index

The PSQI is a self-administered questionnaire that assesses subjective sleep quality over one month^24,25^. It calculates scores for seven factors: sleep quality, latency, duration, efficiency, disturbance, medication use, and daytime dysfunction. Each factor is rated on a 0–3 scale, with a maximum score of 21. Scores of ≥ 6 are indicative of sleep disturbance. The Japanese version of the questionnaire was used in this study. Its validity and reliability has been established in a previous study^25^.

### Statistical analysis

The differences in each subjective and objective sleep parameter, ⊿TST (sj-TST-oj-TST), ⊿SOL (sj-SOL-oj-SOL), and ⊿WASO (sj-WASO-oj-WASO), were calculated as an index of the degree of SSM. For the lights-out time, last awakening time, TIB, and each of the subjective/ objective sleep parameters, the differences between the values for each test day and the previous day were squared, and the mean of the squares of all differences (mean squared successive differences; MSSD) was calculated as an index of the intra-individual variability in each sleep habit and subjective/objective sleep parameter^27,28^. All data were expressed as means and standard deviations. Comparisons of each variable between the insomnia and non-insomnia groups were made using an uncorrelated t-test for normally distributed items and the Mann–Whitney U test for non-normally distributed variables. Pearson's correlation analysis was used to analyze the correlations between bedtime behavior (lights-out and get-up times in the subjective sleep parameters), sleep parameters, and daytime dysfunction in each group. Statistical significance was set at p < 0.05. SPSS (version 28.0) was used to analyze the data.

## Results

### Comparison of insomnia and non-insomnia groups

Table 1 presents the comparison for each variable. Thirty-one participants in the insomnia group and 42 in the non-insomnia group met the inclusion criteria. The insomnia group had a mean age of 63.2 ± 11.7 years, and 19.4% were male. In contrast, the non-insomnia group had a mean age of 59.6 ± 9.4 years, and 38.1% were male. There were no significant differences in the age and sex ratios.

**Table 1:** Comparison of insomnia and non-insomnia groups for each sleep variable and bedtime behavior.

Sleep parameters	Insomnia group	Non-insomnia group	p-value
Sex (M/F)	31(6/25)	42(16/26)	0.085
Age (years)	63.2(11.7)	59.6(9.4)	0.146
sj-TST (min)	319.0(74.3)	381.0(51.5)	< 0.001**
sj-WASO (min)	49.6(52.5)	10.4(13.3)	< 0.001**
sj-SOL (min)	40.9(33.0)	13.2(8.5)	< 0.001**
oj-TST (min)	362.6(63.0)	323.9(55.1)	0.007**
oj-WASO (min)	76.9(42.9)	71.2(39.6)	0.561
oj-SOL (min)	16.0(9.8)	19.3(12.1)	0.219
ΔTST (min)	-43.7(100.3)	57.1(46.2)	< 0.001**
ΔWASO (min)	-27.3(52.7)	-60.8(40.7)	0.003**
ΔSOL (min)	24.8(35.1)	-6.2(12.9)	< 0.001**
Light-off time (h:m.)	23:15(70.7)	23:48(60.8)	0.037*
Get-up time (h:m.)	7:03(62.9)	6:50(62.6)	0.304
TIB (min)	467.5(65.1)	422.8(51.9)	0.002**
MSSD-			
sj-SOL	2498.7(4159.6)	355.9(935.0)	< 0.001**
sj-WASO	3346.5(4176.0)	1090.5(2358.0)	< 0.001**
sj-TST	10,838.8(9679.4)	10,217.3(8959.4)	0.973
oj-TST	5274.5(3962.9)	9322.6(7713.1)	0.006**
oj-SOL	1504.3(1903.5)	1141.5(1761.5)	0.676
oj-WASO	2638.4(2527.3)	2582.4(2151.3)	0.592
oj-TST	5274.5(3962.9)	9322.6(7713.1)	0.006**
Light-off time	1683.4(1911.7)	5403.5(7637.3)	< 0.001**
Get-up time	6126.7(6562.0)	7353.5(9906.8)	0.982
TIB	7362.8(10,880.5)	13,820.4(29,249.3)	0.066
AIS	9.0(5.2)	5.0(3.8)	< 0.001**
SDISS	8.1(6.1)	3.9(4.7)	0.003**
PSQI	11.3(6.6)	5.5(3.2)	< 0.001**

Values are presented as means (and standard deviations) or numbers (%). P-values associated with significant results are labeled with asterisks (p < 0.05) or double asterisks (p < 0.01).

*sj-TST* subjective total sleep time, *sj-WASO* subjective wake-after-sleep onset, *sj-SOL* subjective sleep onset latency, *oj-TST* objective total sleep time, *oj-WASO* objective wake-after-sleep onset, *oj-SOL* objective sleep onset latency, *ΔTST* sj-TST - oj-TST, *ΔWASO* sj-WASO - oj-WASO, *ΔSOL* sj-SOL - oj-SOL, *TIB* total time in bed, *MSSD* Mean squared successive differences, *AIS* Athens Insomnia Scale, *SDISS* Sheehan Disability Scale, *PSQI* Pittsburgh Sleep Quality Index.

### Sleep state and bedtime behavior

The insomnia group reported an sj-TST 62 min shorter (p < 0.001), sj-WASO 39 min longer (p < 0.001), and sj-SOL 28 min longer (p < 0.001) than the non-insomnia group (Table 1). Conversely, the insomnia group reported oj-TST 39 min longer (p = 0.007), oj-WASO (p = 0.561), and oj-SOL (p = 0.219), which were not significantly different from those in the non-insomnia group (Table 1).

The insomnia group had an earlier bedtime than the non-insomnia group (33 min, p = 0.037). However, there was no difference in waking time between the two groups (p = 0.304), resulting in a significantly prolonged TIB (45 min, p = 0.002).

The insomnia group had poorer sleep and daytime dysfunction than the non-insomnia group in regards to AIS (9.0 vs. 5.0 points, p < 0.001), SDISS (8.1 vs. 3.9 points, p = 0.003), and PSQI (11.3 vs. 5.5 points, p < 0.001). The insomnia group exceeded the cutoff scores on all scales.

### Sleep state misperception

**Figure 1:**
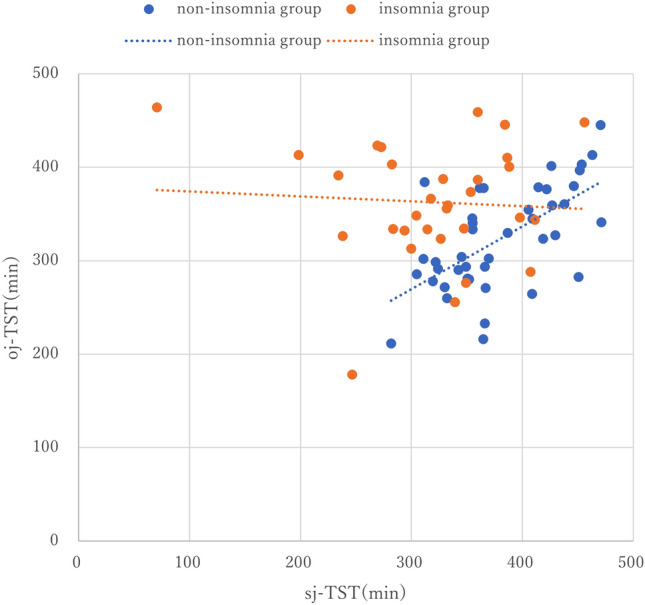
Correlation between subjective and objective total sleep times in the two groups. The vertical axis represents the objective total sleep time, oj-TST, measured using the actigraphy, and the horizontal axis represents the subjective total sleep time, sj-TST, measured using the sleep diary. Orange dots indicate the insomnia group and blue dots indicate the non-insomnia group. The orange dashed line indicates the regression line for the insomnia group and the blue dashed line indicates the regression line for the non-insomnia group. A significant positive correlation was found between sj-TST and oj-TST in the non-insomnia group (R = 0.627, p < 0.001), whereas no correlation was found in the insomnia group (R = − 0.061, p = 0.743). sj-TST subjective total sleep time**,** oj-TST objective total sleep time.

The insomnia group showed significant differences between ⊿TST (-101 min, p < 0.001), ⊿WASO (34 min, p = 0.003), and ⊿SOL (31 min, p < 0.001) compared to the non-insomnia group, indicating that they perceived their sleep state to be worse (Table 1). The non-insomnia group (R = 0.627, p < 0.001) showed a significant positive correlation between the sj-TST and oj-TST, while the insomnia group (R = -0.061, p = 0.743) showed no significant correlation (Fig. 1).

### Intra-individual variability of sleep habits and sleep parameters

Table 1 presents the MSSD in sleep habits and subjective/objective sleep parameters. The intra-individual variability of light-off time was significantly smaller in the insomnia group than in the non-insomnia group (p < 0.001). Similarly, the intra-individual variability of sj-SOL (p < 0.001) and sj-WASO (p < 0.001) was significantly greater in the insomnia group than the non-insomnia group, but those of objective sleep parameters (except for oj-TST, p = 0.006) showed no significant intergroup differences.

TIB length showed a weak but significant positive correlation with the MSSD of sj-SOL (r = 0.372, p < 0.039) in the insomnia group, but not in the non-insomnia group (r = -0.007, p < 0.963). A significant negative correlation was further found between MSSD of lights-out time and AIS score in the insomnia group (r = -0.407, p = 0.031), but not in the non-insomnia group (r = 0.130, p < 0.410).

### Relationship between bedtime behavior and sleep state misperception

The results of the relationship between bedtime behavior and SSM in the insomnia and non-insomnia groups are presented in Table 2. The insomnia group had a negative correlation between TIB and ⊿TST (R = − 0.428, p = 0.016). The longer the bedtime, the greater the underestimation of sleep time. Conversely, there was no significant correlation between the two variables in the non-insomnia group (R = − 0.028, p = 0.859). Similarly, a positive correlation was found between the light-off time and ⊿TST in the insomnia group (R = 0.375, p = 0.038). The earlier the light-off time, the more sleep time was underestimated. In contrast, there was no significant correlation between the two in the non-insomnia group (R = -0.049, p = 0.757).

**Table 2:** Relationship between bedtime behavior and sleep state misperception.

		ΔTST		ΔSOL		ΔWASO	
		R	p	r	p	r	p
Insomnia group	TIB	-0.428*	0.016*	0.292	0.11	0.182	0.327
	Light-off time	0.375	0.038*	-0.306	0.094	-0.181	0.329
	Get-up time	-0.022	0.906	-0.043	0.819	-0.017	0.928
Non-insomnia group	TIB	0.028	0.859	-0.044	0.782	-0.348*	0.024*
	Light-off time	-0.049	0.757	0.167	0.29	0.1	0.529
	Get-up time	-0.025	0.877	0.126	0.425	-0.192	0.224

P-values associated with significant results are indicated by asterisks (p < 0.05).

*sj-TST* subjective total sleep time, *sj-WASO* subjective wake-after-sleep onset, *sj-SOL* subjective sleep onset latency, *oj-TST* objective total sleep time, *oj-WASO* objective wake-after-sleep onset, *oj-SOL* objective sleep onset latency, *ΔTST* sj-TST - oj-TST, *ΔWASO* sj-WASO - oj-WASO, *ΔSOL* sj-SOL - oj-SOL, *TIB* total time in bed.

## Discussion

Using a wearable device that can measure the objective sleep state, this study clarified the actual condition of SSM in patients with chronic insomnia under real-life conditions and extracted the underlying problems in their bedtime behavior. Compared to the non-insomnia group, the insomnia group subjectively underestimated their sleep state despite comparable objective sleep parameters. Specifically, objective WASO and SOL showed no significant group differences, and TST was significantly prolonged in the insomnia group, whereas subjective TST, WASO, and SOL were significantly worse. The better objective sleep parameters in the insomnia group were presumably because they were undergoing pharmacotherapy at a specialized sleep medicine facility. In other words, although there was an objectively noticeable improvement in insomnia symptoms, individuals with the disorder did not subjectively experience satisfaction corresponding to that improvement. The present findings are also consistent with the results of a previous study examining the misperception of actual sleep state in insomnia patients in the home environment^29^.

The fact that the correlation between subjective and objective TST is found only in the non-insomnia group and is lost in the insomnia group is another characteristic of SSM. This is evident in Fig. 1, where a significant number of individuals with insomnia are positioned above the regression line for the non-insomnia group. This indicates that the insomnia group felt the same oj-TST shorter than the non-insomnia group.

Misperceptions of the sleep–wake cycle, sleep-related worry and attention, and brief awakenings have been proposed as possible mechanisms to explain the discrepancy between subjective and objective assessments in patients with insomnia^30^. Several associations with SSM in patients with mood disorders have been reported. The severity of depressive symptoms have been linked to underestimation of the TST^31,32^ and overestimation of the WASO^33^. The insomnia group in this study had a significantly lower SDISS than the non-insomnia group, which was below the established cutoff of 5 that is associated with good sleep health. Such daytime dysfunction may also contribute to the lower subjective ratings of sleep status.

The present results suggest that it is difficult to distinguish between insomnia and non-insomnia groups based on objective sleep parameters and that there is an extensive overlap. Major diagnostic criteria for insomnia, such as DSM-5 and ICSD-3, do not include objective sleep parameters, and their diagnosis is based solely on subjective complaints. Current literature^34^ suggests that insomnia with short objective sleep duration is associated with emotional and cortical arousal, stress response system activation, impaired cardiovascular and metabolic function, neurocognitive impairment, and high mortality risk. However, insomnia with normal objective sleep duration is associated with SSM without stress response system activation or medical complications. The latter is primarily associated with psychological causes of onset, with specific bedtime behaviors that exacerbate insomnia symptoms, such as early bedtime and prolonged bedtime. This type of insomnia responds positively to cognitive behavioral therapy to modify these behaviors.

Furthermore, patients with insomnia and SSM who participated in this study showed bedtime behavioral changes, such as attempting to stay longer in bed than in the non-insomnia group, suggesting that this was related to the severity of SSM. Specifically, a negative correlation was found between TIB and ⊿TST in the insomnia group, indicating that the longer a patient stayed in bed, the greater the degree of underestimation of TST. TIB is the difference between lights-out and get-up time, and sleep parameters tended to worsen as lights-out time increased; this trend was more pronounced in the insomnia group.

Although the findings in this study do not indicate a causal relationship between changes in bedtime behavior and the onset or worsening of insomnia, they are consistent with the concept of sleep restriction and compression in cognitive behavioral therapy for insomnia (CBT-I). Most guidelines list CBT-I as the first-line treatment for insomnia^35–38^.

Prior studies have suggested the existence of significant intra-individual variability in bedtime habits and sleep parameters in patients with insomnia^17,29,39,40^. In the present study, the intra-individual variability (MSSD) of subjective SOL and WASO was significantly higher in the insomnia group than in the non-insomnia group, while the greater intra-individual variability of subjective SOL was positively correlated with the length of the TIB. Conversely, in contrast to the previous study, the intra-individual variability of the light-off time was significantly smaller in the insomnia group, possibly due to the fact that many patients were instructed by their doctors to maintain a constant medication schedule, light-off time, and get-up time. However, it is interesting to note that even with the limitations of such sleep hygiene instruction, a significant negative correlation still existed between greater intra-individual variability in lights-out time and insomnia severity, as indicated by the AIS score. Although this causal relationship is not certain, it is possible that patients with more severe insomnia are more preoccupied with sleep problems and are committed to ensuring a constant bedtime, regardless of whether they are sleepy or not. These findings indicate the instability of insomnia symptoms in patients with insomnia, as well as the underlying inappropriate bedtime habits that are included in diagnostic criteria such as ICSD-3. This idea is also supported by previous studies that observed reduced variability in subjective sleep parameters before and after CBTI^39^.

This study has several limitations. The first is that there was a small number of participants in each group. Additionally, the average age of the participants was around 60 years; therefore, the results of this study may not be directly applicable to younger or older patients. The insomnia groups were taking medication, while the non-insomnia groups were not taking any medication. This asymmetry may affect both subjective and objective data. In addition, although previous studies have reported that subjective sleep duration depends on the amount of slow wave sleep^41^, the present study lacked a detailed examination of the type and amount of oral medications. As a methodological limitation, sleep assessment using actigraphy has several limitations despite its advantages. The mechanical characteristics of actigraphy make it difficult to distinguish between sleep and silent awakening (a state of inactivity while awake) on an electroencephalogram (EEG). This is particularly true for individuals with insomnia who are obsessed with going to bed or taking sleeping pills. In the current study, we expected that subjective and objective TST would coincide in the non-insomnia group; however, contrary to expectations, the non-insomnia group had a shorter oj-TST than sj-TST. This may be due to actigraphy’s-over-detection of body movements during sleep, such as tossing, turning, and arousal. It is also possible that the non-insomnia group had a shorter sleep duration, a so-called sleep debt living situation. The MTN240 has been reported to have an 85% concordance rate with the PSG test in a previous validity assessment^26^. However, the influence of the patient’s disease characteristics and medications cannot be completely ruled out. Finally, the presence of undiagnosed sleep apnea or periodic limb movement disorder was unknown, as PSG was not performed. Although objective sleep parameters by PSG and actigraphy are normal, it has been reported that the presence of SSM can be inferred from the microstructure of electroencephalograms (EEG)^11,12^. However, this could not be evaluated in the present study due to the lack of EEG findings.

In conclusion, patients with chronic insomnia had worse subjective sleep status than healthy participants, causing daytime dysfunction regardless of objective sleep status. Until now, the objective sleep state assessment of SSM has mainly been performed by PSG testing^42^. This study used actigraphy to continuously measure natural sleep habits and states in the home environment, which is difficult with PSG. The severity of SSM in patients with insomnia was associated with an earlier bedtime and longer TIB. The results of this study support the validity of sleep restriction methods in CBT-I. Examining the sleep habits of individuals experiencing insomnia in their home environment and monitoring their sleep status in relation to these habits could be instrumental in identifying key points for intervention and providing targeted guidance on sleep hygiene and CBT-I.

## Data Availability

The data of this study are not publicly available due to the privacy of the participants. The data are available on request from the corresponding author.
